# An easy tool to assess ventilation in health facilities as part of air-borne transmission prevention: a cross-sectional survey from Uganda

**DOI:** 10.1186/s12879-017-2425-6

**Published:** 2017-05-03

**Authors:** Miranda Brouwer, Achilles Katamba, Elly Tebasoboke Katabira, Frank van Leth

**Affiliations:** 1 PHTB Consult, Lovensestraat 79, 5014 DN Tilburg, The Netherlands; 2 Department of Medicine, School of Medicine, Makerere University, College of Health Sciences, P.O. Box 21696, Kampala, Uganda; 3 Amsterdam Institute of Global Health and Development, Pietersbergweg 17, 1100 DE Amsterdam, The Netherlands

**Keywords:** Ventilation, Infection control, Tuberculosis, Uganda

## Abstract

**Background:**

No guidelines exist on assessing ventilation through air changes per hour (ACH) using a vaneometer. The objective of the study was to evaluate the position and frequency for measuring air velocity using a vaneometer to assess ventilation with ACH; and to assess influence of ambient temperature and weather on ACH.

**Methods:**

Cross-sectional survey in six urban health facilities in Kampala, Uganda. Measurements consisted of taking air velocity on nine separate moments in five positions in each opening of the TB clinic, laboratory, outpatient consultation and outpatient waiting room using a vaneometer. We assessed in addition the ventilation with the “20% rule”, and compared this estimation with the ventilation in ACH assessed using the vaneometer.

**Results:**

A total of 189 measurements showed no influence on air velocity of the position and moment of the measurement. No significant influence existed of ambient temperature and a small but significant influence of sunny weather. Ventilation was adequate in 17/24 (71%) of all measurements. Using the “20% rule”, ventilation was adequate in 50% of rooms assessed. Agreement between both methods existed in 13/23 (56%) of the rooms assessed.

**Conclusion:**

Most rooms had adequate ventilation when assessed using a vaneometer for measuring air velocity. A single vaneometer measurement of air velocity is adequate to assess ventilation in this setting. These findings provide practical input for clear guidelines on assessing ventilation using a vaneometer. Assessing ventilation with a vaneometer differs substantially from applying the “20% rule”.

## Background

Tuberculosis (TB) is an airborne disease of which transmission occurs through infectious droplets in the air originating mostly from coughing people. This makes health care facilities high-risk areas for TB transmission because coughing patients, including those with (undiagnosed) TB, gather there when seeking care. Therefore, by the nature of their work, health care workers have an increased exposure to TB, and a higher risk of TB disease compared to the general population [1]. To reduce the risk of TB transmission in health care facilities, the World Health Organization (WHO) recommends a set of TB infection prevention and control measures [2]. These measures include the use of ventilation systems. In existing health care facilities maximizing natural ventilation takes priority before considering other ventilation systems.

Evaluation of the adequacy of ventilation is through assessment of the number of air changes per hour (ACH) [2]. This is the number of times per hour that air from outside the room replaces the air in the room. International guidelines recommend at least 12 ACH for airborne precaution rooms [2, 3], and at least 6-12 ACH for laboratories performing low risk investigations such as smear microscopy [4]. If individual health care workers or health care facilities had a simple tool to assess ventilation in their workrooms, it may encourage them to maximize natural ventilation. If adequate ventilation is not possible, they could use additional measures to reduce the airborne transmission risk.

The most used reference tests for measuring actual ACH are tracer gases or carbon dioxide dilution [5–7], as described by Menzies et al. [8] These techniques require equipment that has limited availability in resource-constrained settings. Other techniques have been used, such as asking health care workers about ventilation in their consultation rooms without quantitative assessment [9], or the open openings’ surface to floor surface ratio to assess ventilation, the “20% rule” [10], as recommended in the Ugandan TB infection control guidelines [11]. Ventilation is considered adequate if the surface of open openings is more than 20% of the floor surface. These methods are easy to use but have not been validated against an adequate reference method.

The document on implementation of the WHO infection prevention and control policy suggests a relative simple tool, a vaneometer, to assess ventilation [12]. The vaneometer is developed for industry to measure air velocity. This air velocity together with the volume of the room and the surface of openings through which air enters the room, provide the inputs to calculate the ACH.

Unfortunately, there is no operational guidance nor experiences from published studies on how to measure air velocity using the vaneometer, precluding the answers to some basic questions such as (1) Is a single air velocity measurement sufficient, and (2) Is the position in the opening relevant for the air velocity measurement? For widespread implementation of ventilation assessments in especially resource limited settings it would be of great help if a single measurement of air velocity would suffice, which is the primary research question for the current study. Assessment of ventilation does need trained staff, and if a single measurement were sufficient, staff could perform more assessments and cover more facilities in less time. A secondary question is how the ACH assessment with vaneometer compares to the assessment of the open openings’ surface to floor surface ratio method.

## Methods

In six purposefully chosen urban health care facilities in Kampala, we conducted ventilation assessments in the TB clinic, the laboratory, an outpatient department (OPD) consultation room, and in the OPD waiting area. Data collectors took nine rounds of separate air velocity measurements for each opening using a vaneometer: three times a day on three consecutive working days. At each of these time points, they took the measurements at five positions in each opening in the room: in the center of the opening and in the middle of each of the sides of the opening. They kept the vaneometer for a few seconds at each position and then read the air velocity. The measurements were taken with openings open or closed as in routine working conditions.

In addition, they measured the height and width of all openings to calculate the surface of the openings, as well as width, length and height of the rooms to calculate the volume of the rooms. They recorded information on ambient temperature (degrees centigrade) and weather conditions (cloudy, rainy, sunny, windy or a combination of these) at the time of the measurement. The recording of open or closed state of the openings as in routine working conditions occurred on the first day only.

The data collectors used an android phone with pre-installed structured data capture forms using Open Data Kit Collect (version 1.4.2.). The forms were uploaded using Open Data Kit Aggregate to a server from which databases in the form of comma separated files were downloaded. The data collectors received training on the use of the vaneometer and had prior experience conducting such assessments. They used a DwyerTM vaneometer M480 with a vane (Dwyer Instruments, Inc., Michigan City, USA) to measure air velocity in meters per second. The selection for this type of vaneometer was based on the price (USD 35,75 at the time of the study) and the experience that researchers and data collectors had with this type. The air velocity lower detection limit of this device is 25 ft per minute or 0.13 m per second (manufacturer instructions leaflet).

### Analysis

The data files were imported into STATA version 12 (StataCorp, College Station, Texas, USA). To assess the appropriateness of the use of the vaneometer, we estimated the effect of the position of the measurement and the round of the measurement of air velocity at a specific opening. We used a hierarchical model that incorporated a fixed effect for the round, and a random effect for the position of the measurement. The fixed effect of round denotes how much the mean overall velocity changes on average for each round. The random effect allows the mean overall velocity to differ by opening. Its estimate is a standard deviation and consists of two parts: a between-estimate and a within-estimate. The between-estimate gives the standard deviation of the different mean overall velocities at each position. A small between-random effect indicates that there is not much variation in overall mean velocity between the different positions. The within-estimate gives us the standard error of the actual measurements as is similar to the residuals in every statistical model. The difference in magnitude of these parts of a random effect tells us where the variation in velocity measurement comes from.

As input for the model, we used only measured air velocities that had an inward direction and were not equal to zero. The estimated air velocities for each opening provided the input for the formula of ACH

$$ ACH=3600\;\mathrm{s}\; x\frac{\left( average\kern0.5em  estimated\kern0.5em  air\kern0.5em  velocity\kern0.5em \left( m/ s\right)\right. x\left( area\  all\  openings\  with\  incoming\  air\ (m2)\right)}{volume\  of\  the\  room\ (m3)} $$

$$ \mathrm{s}=\mathrm{seconds},\mathrm{m}/\mathrm{s}=\mathrm{meter}\ \mathrm{per}\ \mathrm{second},\mathrm{m}2=\mathrm{square}\ \mathrm{meter},\mathrm{m}3=\mathrm{cubic}\ \mathrm{meter} $$

If the air velocity in an opening was not inward for all five positions, the area of the opening with inward air contributed proportionally to the ACH calculation. For example, if the direction of the airflow was inward in three of the five positions and outward in the remaining two positions, 60% of the total area of the opening contributed to the ACH calculation. We classified ventilation as inadequate if the ACH was below 6, as potentially adequate between 6 and 12, and as adequate if above 12 [2–4].

To assess the effect of weather, we collapsed the possible categories into two (sunny / not sunny) to obtain groups of similar sizes. Given the distribution of temperature, we grouped the data as below 25 degrees or 25 degrees and over.

We calculated the open openings’ surface to floor surface ratio with R statistics [13].

The “20% rule” uses the formula

$$ ventilation=\frac{sum\; of\  the\  surface\  of\; all\; open\  open ings}{surface\  of\  the\  floor\  of\  the\  room} x\;100\% $$

The assessment of ventilation using the minimum ACH value calculated with the measured air velocity was then compared to the assessment of the ventilation with the “20% rule”.

### Ethics

The Research and Ethics Committee of Makarere University and the Uganda National Council for Science and Technology in Kampala approved the Ugandan study.

## Results

Data collection took place from May to July 2014. In the six facilities, the data collectors took 189 measurements, i.e. measuring the air velocity at each opening in the room, out of the expected 216 (six facilities, four rooms, and nine rounds of measurements: three times a day on 3 days). Two TB clinics were tents, which were completely open structures with a roof and poles only. In one of them we took measurements on 1 day only, in the other TB tent no measurements at all. In one facility, we managed only 2 days of measurements. In one room in two facilities we did not manage three rounds of measurements in a day because the rooms were in use. In total, there were 3955 air velocity measurements, of which 278 (7%) were zero.

The effects of the hierarchical model are reported in Table 1. The average fixed effect of the round on the measured air velocity at a specific opening was small in relation to the mean overall air velocity at that opening, even if this effect was statistically significant. The between part of the random effect of the position of the measurement was in most instance almost non-existent, and always much lower than the within random effect. These results indicate that a single measurement at an arbitrary position of the opening would give a valid indication of the air velocity at that opening. Using these measurements in the calculation of the ACH would provide a valid assessment of the ventilation in the room.

**Table 1:** Effects of the hierarchical model

Facility	Room	Mean overall velocity	Average effect round			Random effect position	
			Estimate	95% CI	*p*-value	Between^*^	Within
Facility 1	Laboratory	0.36	−0.01	−0.03 - 0.05	0.185	3.45 e-14	0.21
	OPD consultation	0.49	0	−0.03 - 0.04	0.827	3.03 e-10	0.38
	OPD waiting room	0.27	−0.01	−0.02 – 0.00	0.043	1.26 e-13	0.16
	TB room	0.19	0.01	−0.01 - 0.02	0.435	1.36 e-13	0.17
Facility 2	Laboratory	0.22	0.03	0.00 - 0.07	0.024	2.96 e-08	0.41
	OPD consultation	0.16	0.01	−0.01 - 0.02	0.284	5.22 e-11	0.11
	OPD waiting room	0.22	0.02	0.00 - 0.03	0.025	0.13	0.22
	TB room	0.17	0.01	−0.01 - 0.03	0.069	0.02	0.12
Facility 3	Laboratory	0.15	0	−0.01 - 0.00	0.207	6.39 e-15	0.08
	OPD consultation	−0.31	−0.02	−0.04 - 0.00	0.016	4.42 e-11	0.16
	OPD waiting room	0.38	0.01	−0.00 - 0.02	0.372	5.04 e-10	0.34
	TB room	0.39	0.11	−0.02 - 0.25	0.083	1.09 e-13	0.3
Facility 4	Laboratory	0.21	0.01	0.00 - 0.03	0.089	1.59 e-09	0.2
	OPD consultation	0.52	0.04	−0.01 - 0.1	0.104	5.25 e-11	0.41
	OPD waiting room	0.37	0.03	0.0 - 0.05	0.048	0.02	0.36
	TB room	0.23	0.02	0.00 - 0.04	0.045	2.01 e − 12	0.24
Facility 5	Laboratory	0.29	0.06	−0.01 - 0.127	0.071	4.60 e-13	0.35
	OPD consultation	0.37	0.04	−0.1 - 0.09	0.124	1.05 e-11	0.32
	OPD waiting room	0.25	0.01	−0.01 - 0.03	0.427	3.07 e-10	0.2
Facility 6	Laboratory	0.16	0.01	0.00 - 0.02	0.09	2.05 e-12	0.15
	OPD consultation	0.42	−0.01	−0.04 - 0.01	0.346	4.89 e-13	0.2
	OPD waiting room	0.24	0.03	0.01 - 0.04	< 0.001	0.04	0.3
	TB room	0.25	0.01	−0.02 - 0.03	0.596	0.06	0.2

^*^Scientific notation y e-14 meaning y^−14^

The model presented in this table included the adjusting variables weather and temperature, which are not shown in the table

Table 2 presents the classification of ACH based on the modeled air velocity and the 20% rule. In 17 of the 23 (74%) rooms, all rounds of measurements conducted resulted in adequate ventilation. In one room, only one round resulted in inadequate ventilation, while all other rounds in the same room resulted in potentially adequate or adequate ventilation. The other six rooms had a combination of potentially adequate or adequate ventilation.

**Table 2:**
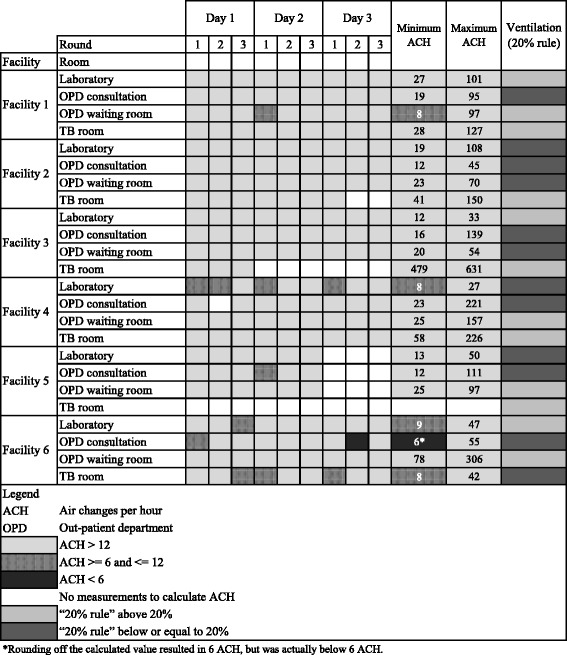
Ventilation status in four areas in six urban health care faculties in Uganda per round of measurement and per day

The modeled air velocity did not vary significantly with the ambient temperature (*p* = 0.259). In sunny weather the air velocity was higher compared to non-sunny weather (*p* = 0.003), though the difference in the mean estimated air velocity in both weather conditions was rather small (0.07 m/s), meaning that under different weather conditions the air velocity may change. Another single measurement would be needed to assess the ACH under the different weather conditions.

**Fig. 1:**
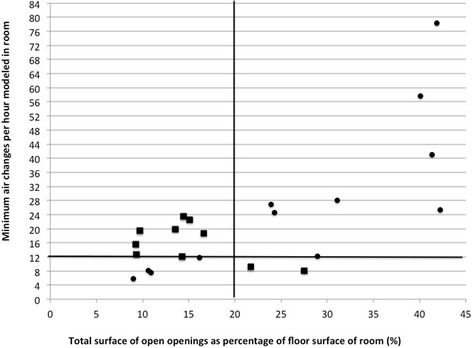
Scatter plot of minimum ACH and the 20% rule in each room. *Black squares* show roooms where both methods did not agree (■) and *black dots* show rooms where both methods did agree on the ACH assessment (•). The value of the minimum ACH in the TB room in facility 3 was excluded from the scatterplot because of its high value (479) it distorted the plot

The ventilation in the routine working situation with the “20% rule” showed that 12 of the 24 rooms assessed had a ratio of more than 20%, which is considered adequate ventilation (Table 2). Agreement between the two methods existed in 13/23 (56%) of the rooms if we combine the potentially adequate and adequate ventilation categories of the ACH method into one category of adequate ventilation. In one room we did not have air velocity measurements and therefore could not make the comparison. In Fig. 1 we show the two methods in a scatterplot where the left upper quadrant and the right lower quadrant show assessments where the methods did not agree.

## Discussion

Our results suggest that in this setting a single air velocity measurement at all openings in a room using a vaneometer is sufficient to assess ventilation in that room through the calculation of ACH. Ventilation assessed with the vaneometer was classified as adequate in most of the rounds. These findings do not compare well with the “20% rule” because both methods agreed in only 56%.

The weather condition had a rather small effect on the measured air velocity and may be due to a difference in the temperature gradient between in- and outside temperature. Because we did not measure outside temperature we cannot verify this. However, the effect was rather small on the estimated air velocity and will probably not affect the ACH. Though different weather conditions may affect the opening of windows and doors compared to the routine working situation, which would affect ACH. Therefore, we recommend assessment of ACH under various weather conditions to verify the ventilation in this different condition.

The finding of (potentially) adequate ventilation in more than 94% (177/189) of the rounds was surprising. We did expect poorer ventilation based on other studies from Africa reporting less than 50% of rooms adequately ventilated, though with a different assessment method [10, 14]. The “20% rule” ventilation assessment of 50% adequately ventilated rooms agreed with another study from Uganda [10]. Deciding on the most appropriate assessment of ventilation systems would require a validation study using for example tracer gases.

We used 12 ACH as cut-off for adequate ventilation. This cut-off recommendation applies to mechanically ventilated airborne precaution rooms [2]. The recommended cut-off for laboratories is 6-12 ACH [4]. No clear recommendations on ACH exist for the other rooms such as TB clinics, OPD consultation and waiting rooms, or wards. In a systematic review, Li et al. did not find evidence for a recommended quantification of ventilation requirements [15]. A study in Canada found an association between general or non-isolation rooms having less than 2 ACH and the conversion of the tuberculin skin test in health care workers [16]. The study did not find an association between skin test conversion and inadequately ventilated isolation rooms for which at the time of the study the cut-off was 6 ACH. If a lower cut-off of more than 6 ACH instead of more than 12 ACH would be acceptable to define adequate ventilation, only one room in one round in Uganda would have inadequate ventilation.

Natural ventilation has been shown to achieve higher ACH than mechanical ventilation [5, 7]. The disadvantage of natural ventilation is its variability in both velocity and direction [17]. However, given the costs of mechanical ventilation systems and the need to maintain these systems, and the weak evidence available for specific recommendations regarding the quantification of ventilations requirements, natural ventilation seems the way forward for resource limited settings. Our study shows that in Uganda natural ventilation provides adequate ventilation in at least 50% (“20% rule”) or 71% (vaneometer) of the facilities and rooms assessed.

Our method is easy and simple to use and provides a rough estimate of the ACH. It will give health care workers an idea whether their place of work is probably safe with regard to ventilation as prevention for air-borne transmission. However, if the assessment needs to be precise because of working with high risk patients such as patients with MDR-TB, then a rough estimate is insufficient.

Health facilities would need practical guidelines to assess ventilation using the vaneometer in their rooms. Based on our findings, not validated by a reference method, we suggest that such practical guidelines could include at least the following items:

A single measurement of air velocity at each opening using a vaneometer and measurements of openings and rooms provides adequate input for the ACH calculation;

If ACH is above 12 the ventilation is deemed adequate;

If the ACH is between 6 and 12, several measurements of air velocity provide insight into the variability of ventilation; if persistently between 6 and 12, opening more openings will probably increase ventilation;

Because of a potential effect of the weather, assessment of the ACH under different weather conditions is necessary;

If opening of more openings is not possible, or the ACH is below 6, then health facility management should consider improving health care worker safety through additional measures for infection prevention and control; and.

Training and support for ventilation assessments: infection control officers could conduct the assessments after a practical training on how to measure air velocity and how to calculate ACH.

Additional measures to reduce the TB transmission risk in rooms with inadequate ventilation assume that all administrative controls are in place [2]. Additional measures include positioning of health care workers such that they would not inhale potentially infected air, and fans to direct airflow out of the room. Construction adaptation such as addition windows to allow cross-ventilation or latticed walls, seem most effective, though not easily implemented [18]. Each situation with inadequate ventilation would need individual assessment on how to improve ventilation in the particular circumstances of that situation. Should all these measures be insufficient to contain the transmission risk health care workers may need to wear particulate respirators. To do that effectively, they need clear instructions on how and when to use these and how to handle the respirators in-between use should the respirators be used more than once [19].

### Limitations

This method of ACH calculation assumes perfect mixing of air in the entire room. This may not happen in rooms that have obstacles such as partition walls or patient screens. Imperfect mixing means that some areas in the room are better ventilated than other areas.

A further limitation to this study is that, in common with many resource-constrained settings, we lacked the resources to validate the vaneometer against a reference test for ACH assessment using trace gases [20] or carbon dioxide dilution [5, 7]. Such validation is urgently needed. Until such research is done, our findings should be interpreted cautiously.

We did not measure outside wind speed, which has been shown to influence ACH [7]. Therefore future research should also measure ambient, outside wind speed and test the extent to which this influences vaneometer assessment of natural ventilation ACH. For example, on still days, with little wind, airflow through room openings may be too low to measure with the vaneometer, possibly causing ACH to be under-estimated.

Although the manufacturer instructions for the vaneometer states accuracy to ±10% of the full scale, the reading of vaneometer is not straightforward because of the constant movement of the vane. However, the data collectors were trained and experienced in taking the readings as such minimizing reading variability. This inter-reader variability potentially results in different assessments of the ventilation in a room, and becomes especially important when the resulting ventilation is below 6 ACH. We therefore recommend taking more than one air velocity measurement if the resulting ACH is between 6 and 12.

In addition, the lower detection limit of the vaneometer device may assess ACH insufficiently in situations with low air velocity.

We assessed the area of the open openings only on the first day, which limits the comparison between the ACH assessment with the “20%-rule”. Data collection took place on three consecutive working days, which may have resulted in the same openings being open or closed during all measurements, it would have been better to assess this at reach round of measurements.

Our study does not capture the complexity of ventilation that is influenced by many factors such as in- and outside temperature and surrounding structures. This was on purpose because we wanted to assess ventilation with simple to use tools and methodology which can be used in the many health facilities in settings with limited resources where more complicated ventilation assessment methods are not widely available. Also, the technical expertise to do such assessment is not or limited available. Our proposed method is easy to implement after a short training and provides a reasonable assessment of the ventilation status. Though we consider a single measurement sufficient for assessing ventilation, we do acknowledge that this method needs further validation. This method is probably of less value in situations where good infection control is highly important such as places where patients with MDR-TB receive treatment. However, it can provide an initial assessment that informs policy makers for further requirements.

## Conclusion

It seems possible to assess ventilation in rooms in health care facilities using a vaneometer taking a single measurement of air velocity at each opening in the rooms. Further studies need to validate our findings and identify simple to use and implement methods to assess ventilation in the many health facilities in limited resources settings with a potentially high prevalence of airborne transmitted diseases such as TB. Such studies would provide further valuable input for guideline development on how to assess ventilation in health care facilities. These studies would also need to assess the usefulness and place of the “20% rule”. An application for mobile phone to facilitate the ACH calculation and one for using the “20% rule” would simplify the assessment even further.

## Abbreviations

ACH: Air Changes per Hour
OPD: Outpatient Department
TB: Tuberculosis
USD: United States Dollar
WHO: World Health Organization
